# Comprehensive elaboration of circular RNA in multiple myeloma

**DOI:** 10.3389/fphar.2022.971070

**Published:** 2022-08-29

**Authors:** Chunsheng Zhu, Aoxiang Guo, Bao Sun, Zheng Zhou

**Affiliations:** ^1^ Department of Chinese Medicine, The First Affiliated Hospital of Zhengzhou University, Zhengzhou, China; ^2^ Department of Pharmacy, The Seventh Affiliated Hospital, Sun Yat-sen University, Shenzhen, China; ^3^ Department of Pharmacy, The Second Xiangya Hospital, Central South University, Changsha, China; ^4^ Institute of Clinical Pharmacy, Central South University, Changsha, China; ^5^ National Clinical Research Center for Metabolic Diseases, The Second Xiangya Hospital, Central South University, Changsha, China

**Keywords:** CircRNAs, multiple myeloma, drug resistance, promising biomarkers, diagnosis and targeted therapy

## Abstract

Circular RNAs (circRNAs), a novel category of endogenous non-coding RNAs, are usually well conserved across different species with a covalent closed-loop structure. Existing and emerging evidence confirms that circRNAs can function as regulators of alternative splicing, microRNA and RNA-binding protein sponges and translation, as well as gene transcription. In consideration of their multi-faceted functions, circRNAs are critically involved in hematological malignancies including multiple myeloma (MM). In particular, circRNAs have been found to play vital roles in tumor microenvironment and drug resistance, which may grant them potential roles as biomarkers for MM diagnosis and targeted therapy. In this review, we comprehensively elaborate the current state-of-the-art knowledge of circRNAs in MM, and then focus on their potential as biomarkers in diagnosis and therapy of MM.

## Introduction

Multiple myeloma (MM), the second most common hematological malignancy that originates in the bone marrow (BM), is characterized by multifocal proliferation of plasma cells and remains life-threatening and incurable (Röllig et al., 2015; Voelker, 2019). In 2020, there are estimated 176,404 new cases of MM and 117,077 cases of MM-related death (Sung et al., 2021). Although there is an indispensable role played by genetic and epigenetic aberrations, as well as chromosomal instability in MM progression, relapse and drug resistance (Morgan et al., 2012; Sansregret et al., 2018; Wallington-Beddoe et al., 2018), the pathogenesis of MM still remains not fully understood. Furthermore, even though the outcome of MM patients can be improved by the emergence of the targeted drugs, such as proteasome inhibitors and immune modulators, the treatment failure and relapse usually occur. Hence, it is urgent to explore more effective and specific approaches for MM diagnosis and treatment.

Circular RNAs (circRNAs), a peculiar group of noncoding RNAs widely existing in mammalian cells, were initially found in 1976 and identified as ‘‘covalently closed circRNAs molecules” (Sanger et al., 1976; Slack and Chinnaiyan, 2019; Huang et al., 2020). Recently, circRNA has become a research hotspot due to its function to regulate multiple processes that include alternative splicing, microRNA and RNA-binding protein sponges, as well as transcriptional and posttranscriptional gene expression (Chen, 2016; Pamudurti et al., 2017; Kristensen et al., 2019; Verduci et al., 2019). To date, increasing literature demonstrates that circRNAs play a crucial role in the pathology of various diseases, including neuronal diseases (Kumar et al., 2018; Dube et al., 2019), diabetes (Liu et al., 2019) and cancer (Guarnerio et al., 2016b; Tang et al., 2021). Particularly, circRNAs serve important functions in hematological malignancies (Bonizzato et al., 2016; Dahl et al., 2018), and our group also reveals that circRNAs can be a promising prognostic biomarker in hematological malignancies (Zhong et al., 2021). Furthermore, circRNAs have also been found to be critically involved in cancer initiation, development and drug resistance (Guarnerio et al., 2016a; Borran et al., 2020). A variety of circRNAs have been identified to be aberrantly regulated and may serve as functional biomarkers in MM with high-throughput technologies and bioinformatics (Zhou et al., 2020). Thus, in-depth studies of circRNAs will not only increase our knowledge of the mechanisms underlying MM, but also be helpful in exploring MM-related circRNAs as biomarkers or targets for the treatment of MM.

In this review, we summarize the current state-of-the-art knowledge of circRNAs in MM, aiming to provide potential biomarkers and therapeutic targets for MM early diagnosis, prognosis and effective treatment.

## Biogenesis and biological functions of circRNAs

### Biogenesis of circRNAs

**FIGURE 1:**
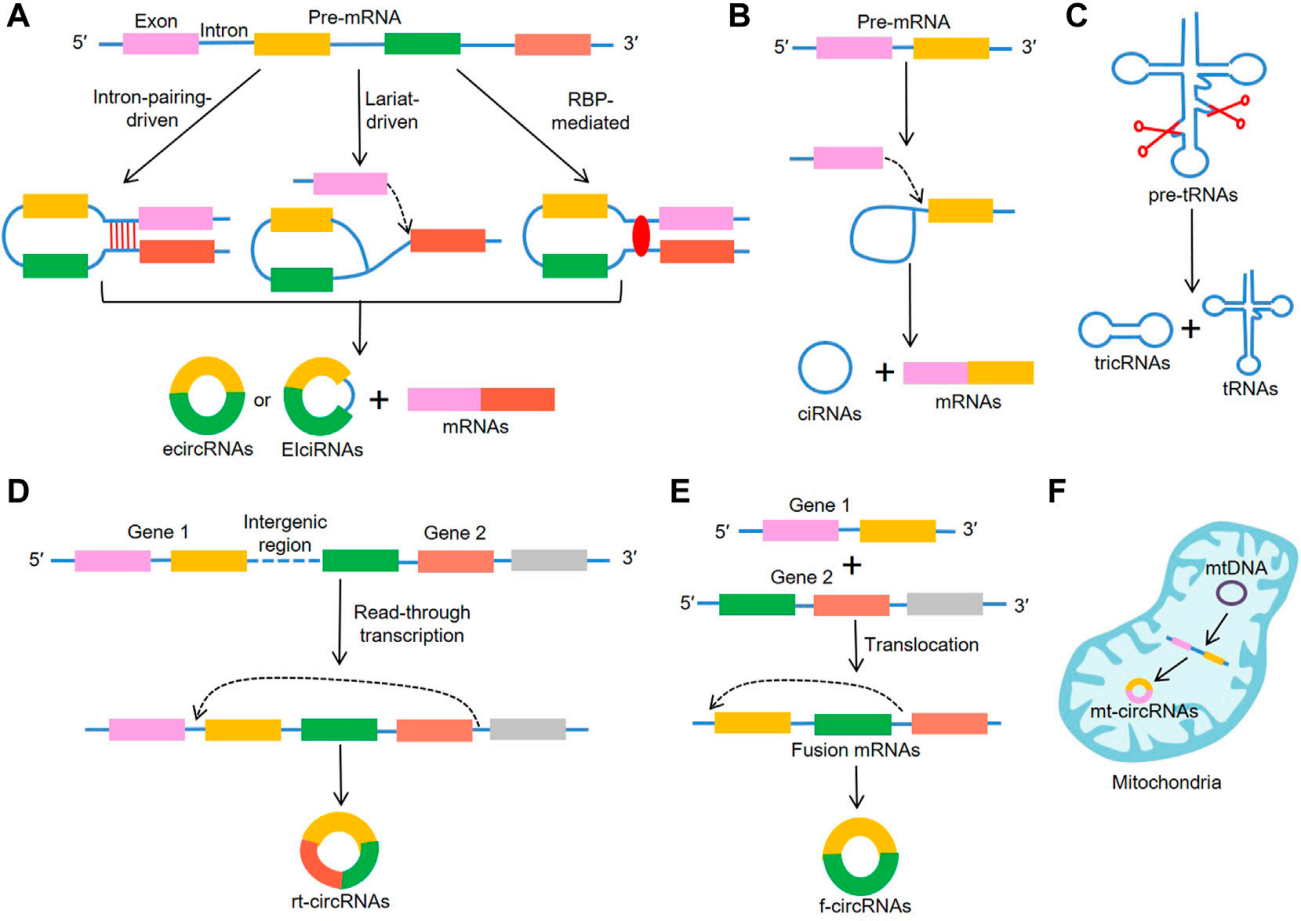
Biogenesis of circRNAs. **(A)** EcircRNAs or EIciRNAs can be generated by “intron-pairing-driven circularization”, “lariat-driven circularization”, “RNA-binding proteins (RBP)-mediated circularization”, **(B)** ciRNAs are generated from intronic lariat precursors that escape the debranching step of canonical linear splicing, **(C)** tricRNAs are generated during the splicing of pre-tRNA, **(D)** rt-circRNAs are from exons between neighboring genes on the same strand (intrachromosomal chimaeras), **(E)** f-circRNAs are fused exons between two distant genes (interchromosomal chimaeras), **(F)** mt-circRNAs are from mitochondrial genomes.

CircRNAs are generally considered to be derived from canonical splice sites and depend on alternative back-splicing. Meanwhile, circRNAs are generated by back-splicing process that ligates a downstream splice-donor site with an upstream splice-acceptor site to form a single-strand covalently closed loop (Chen and Yang, 2015; Holdt et al., 2018). Based on their features and components circRNAs can be mainly classified into three types: exonic circRNAs (ecircRNAs) (Chen et al., 2015) that account for over 80% of all known circRNAs and are predominantly localized in the cytoplasm; circular intronic RNAs (ciRNAs) (Zhang et al., 2013) and exon–intron circular RNAs (EIciRNAs) (Li Z. et al., 2015) that are mainly found in the nucleus. To date, the detailed mechanisms for circRNA biogenesis remain not completely elucidated and there are mainly three proposed models of the formation of circRNAs, including “intron-pairing-driven circularization”, “lariat-driven circularization”, “RNA-binding proteins (RBP)-mediated circularization” (Figure 1A). “Intron-pairing-driven circularization”, where base pairing of Alu repeats or reverse complementary sequences located in the upstream and downstream introns can enhance back-splicing to form the loop (Ivanov et al., 2015). “Lariat-driven circularization” depends on exon skipping and intron lariat formation, where the spliceosomes are gathered to facilitate the connection between 3′ end of an exon and the 5′ end of the same exon or the upstream exon (Barrett et al., 2015; Kelly et al., 2015). Then, the lariat removes the internal intron sequences to release ecircRNAs or EIciRNAs. “RBP-induced circularization”, where RBPs (e.g. Quaking and Muscleblind) regarding as trans-acting factors can enhance circularization by bridging related intronic sequences (Figure 1B) (Conn et al., 2015; Kramer et al., 2015). In addition to the abovementioned models, tRNA intronic circRNAs (tricRNAs) are another type of circRNAs generated during the splicing of pre-tRNA (Figure 1C) (Lu et al., 2015). During the translocation process, read-through circRNAs (rt-circRNAs) are a novel type of circRNAs from exons between neighboring genes on the same strand (intrachromosomal chimaeras) (Figure 1D) (Vo et al., 2019; Vidal, 2020), and fused circRNAs (f-circRNAs) are fused exons between two distant genes (interchromosomal chimaeras) (Figure 1E) (Guarnerio et al., 2016b; Liu et al., 2017; Babin et al., 2021). Recently, mitochondrial genome-derived circRNAs (mt-circRNAs) are discovered, and they seemingly exhibit novel biological characteristics different from nuclear genome-derived circRNAs (Figure 1F) (Liu et al., 2020c; Wu et al., 2020).

### Biological functions of circRNAs

**FIGURE 2:**
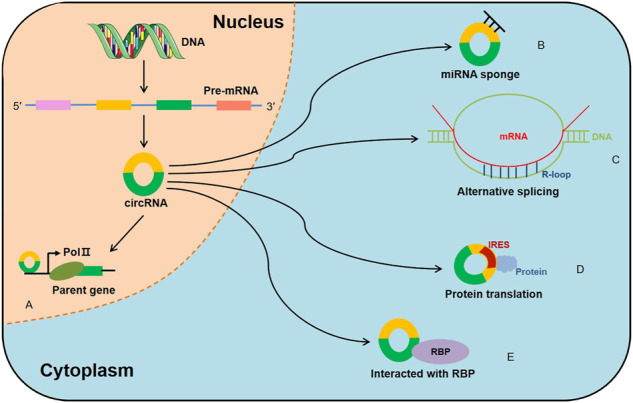
Biological functions of circRNAs. **(A)** CircRNAs regulate the transcription of their parent genes, **(B)** CircRNAs can act as miRNA sponges, **(C)** CircRNAs regulate the alternative splicing through R-loop formation, **(D)** CircRNAs contain IRES elements and mediate the translation of the protein, **(E)** CircRNAs can interact with RBPs to regulate their structure and activity.

Increasing evidence has focused on circRNAs’ biological functions, which include serving as microRNA (miRNA) sponges, modulating alternative splicing and transcription, translation, as well as interacting with RBPs (Figure 2). Generally, ecircRNAs are mainly abundant in the cytoplasm and involved in transcriptional and post-transcriptional control. It has been reported that circRNAs contain complementary miRNA binding sites and serve as miRNA sponges (Piwecka et al., 2017). Due to their high stability, circRNAs are able to accumulate in cells and regulate miRNA to ensure the efficiency of the targets (Thomas and Sætrom, 2014; Huang et al., 2020). CiRS-7 was first reported to serve as miR-7 sponges (Hansen et al., 2013) and was subsequently proved to participate in the pathophysiology of cancer (Yu et al., 2016; Pan et al., 2018). On the other hand, ciRNAs were reported to bind to the Pol II transcription compound to promote the transcription of their parent genes (Zhang et al., 2013). Moreover, EIciRNA interacted with U1 snRNP and promoted transcription of their parental genes (Li Z. et al., 2015). Backsplicing of circRNAs could compete with linear splicing of pre-mRNAs for splicing sites. For instance, circSEP3, was identified to regulate the alternative splicing of its cognate mRNA through R-loop formation (Conn et al., 2017). CircRNAs were also shown to be involved in protein translation. A previous study suggested that circRNAs could mediate the translation of the protein by inserting an internal ribosome entry sites (IRES) in upstream of the start codons of a protein (Wang and Wang, 2015). For example, circ-SHPRH regulated the expression of SHPRH-146aa protein, which protected SHPRH from degradation by the ubiquitin proteasome and ultimately inhibited glioma tumorigenesis (Zhang et al., 2018). Recently, it is demonstrated that certain circRNAs can bind to RBPs through specific binding sites and mediate their maturation, translation and degradation. For instance, circANRIL repressed pescadillo homologue 1 (PES1)-mediated rRNA maturation by binding to PES1 (Holdt et al., 2016). Moreover, circPABPN1 bound to HuR, which reduced the translation of PABPN1 by suppressing HuR from interacting with PABPN1 mRNA (Abdelmohsen et al., 2017). Interestingly, certain circRNAs serving as protein transporters could transport proteins from nucleus to cytoplasm, mitochondria or even membrane (Yang et al., 2017; Wang et al., 2019; Liu et al., 2020c; Du et al., 2020). Collectively, these results indicate that there are more biological functions of circRNA than previously reported, which will be described in further detail in the future.

## CircRNAs in MM

Given the high stability, abundance and conservation, as well as tissue- and developmental-stage-specific expression of circRNAs in human serum, plasma and cells (Li Y. et al., 2015; Memczak et al., 2015), circRNAs display great potential as diagnostic and prognostic biomarkers (Allegra et al., 2022).

### CircRNAs as diagnostic and prognostic biomarkers for MM

In recent years, comprehensive profiling of circRNAs was performed to reveal potential diagnostic and prognostic biomarkers in MM with high-throughput sequencing technologies and bioinformatics (Zhou et al., 2020; Jakobsen et al., 2021) (Table 1).

**TABLE 1:** CircRNAs as diagnostic and prognostic biomarkers for MM.

CircRNAs	Sample	Dysregulation	Potential function	References
Circ-PTK2 and circ-RNF217	64 MM patients and 34 healthy controls	Upregulated	Prognosis	Zhou et al. (2020)
Circ-AFF2	64 MM patients and 34 healthy controls	Downregulated	Prognosis	Zhou et al. (2020)
Circ_0000190	86 MM patients and 30 healthy donors	Downregulated	Prognosis	Xiang et al. (2021)
Circ_101,237	143 MM patients, MM cells and bortezomib-resistant MM cell lines	Upregulated	Diagnosis and prognosis	Liu et al. (2020b)
Circ-MYBL2	89 MM patients and 23 healthy donors	Downregulated	Diagnosis	Yu et al. (2021)
CircMYC	122 MM patients and 54 healthy controls	Upregulated	Diagnosis and prognosis	Luo and Gui, (2020)
Circ_0069767	66 MM patients and 21 healthy controls	Upregulated	Prognosis	Chen et al. (2020a)
Circ_0001821	115 MM patients and MM cell lines	Upregulated	Diagnosis	Liu et al. (2021b)
Circ-ATP10A	20 MM patients and 5 healthy donors	Upregulated	Prognosis	Yu et al. (2022)
Circ_0087776	136 MM patients and 74 healthy controls	Downregulated	Diagnosis	Gong et al. (2021)

*CircRNAs*, Circular RNAs; *MM*, multiple myeloma.

By analyzing circRNA expression in training (4 MM patients and 4 healthy controls) and validating set (60 MM patients and 30 healthy controls), Zhou et al. (Zhou et al., 2020) identified that circ-PTK2 and circ-RNF217 were upregulated and correlated with poor treatment response and survival, while circ-AFF2 was downregulated and associated with good treatment response and survival in MM patients, which might make them potential prognostic biomarkers in MM. Another cohort study enrolling 86 MM patients and 30 healthy donors uncovered that circ_0000190 was decreased and negatively correlated with miR-767-5p in MM patients (Xiang et al., 2021). Meanwhile, circ_0000190 high expression was correlated with better progression-free survival (PFS) and overall survival (OS). These results implied that circ_0000190 and its target miR-767-5p might serve as a prognostic biomarker in MM. A similar study performed by Liu et al. (Liu et al., 2020b) unveiled that circRNA_101237 was significantly upregulated in the bone marrow tissues from 143 MM patients, MM cells and bortezomib-resistant MM cell lines, and it was negatively related to poor prognosis of the patients. Furthermore, overexpressing circRNA_101237 was associated with less responsiveness of MM to bortezomib treatment. Bioinformatic analysis showed that circRNA_101237 might interact with miRNA and mRNA, and further analysis revealed that circRNA_101237 might be involved in signaling pathways, such as PI3K-Akt signaling pathway and chemokine signaling pathway. Therefore, circRNA_101237 may be also a novel diagnostic and prognostic biomarker in MM. Circ-MYBL2, a MM-associated circRNA, was reported to be dramatically decreased in MM tissue and serum samples; simultaneously, low serum circ-MYBL2 was closely associated with unfavorable outcome and had an excellent accuracy in diagnosing MM, which granted its potential as biomarker for MM patients (Yu et al., 2021). Intriguingly, the expression of circulating exosomal circMYC isolated from 122 patients with MM was significantly increased compared with 54 healthy controls (Luo and Gui, 2020); meanwhile, its expression was also significantly higher in bortezomib-resistant patients than that in non-resistant patients. Additionally, higher exosomal circMYC level was linked to higher relapse or mortality rates, suggesting that circulating exosomal circMYC might serve as a biomarker for the diagnosis and prognosis of MM (Luo and Gui, 2020). The expression level of circ_0069767 was detected in 66 samples from MM patients and 21 normal controls, and the results showed that its expression was significantly higher in MM patients (Chen et al., 2020a). Furthermore, MM patients with high expression of circ_0069767 had longer PFS and OS, which could provide a reliable prognostic biomarker for MM patients (Chen et al., 2020a). Liu et al. found that the expression of circ_0001821 was increased in 115 bone marrow tissues of MM patients and MM cell lines compared with that in paired normal controls, and high circ_0001821 expression not only was correlated with poor OS, but also contributed to increased proliferation and apoptosis of MM cells, implying the possibility of circ_0001821 as a new diagnostic biomarker in MM (Liu L. et al., 2021). Recently, Yu et al. (Yu et al., 2022) screened 2052 circRNAs among 20 MM patients and 5 healthy donors and found that the expression of exosomal circ-ATP10A was remarkably increased in MM patients, indicating that it might be a valuable prognostic biomarker in MM.

Although a plethora of biomarkers had been found for the diagnosis of MM, there was still a lack of such specific biomarkers. Several tumor markers for combined diagnosis might be an optimized selection for the accurate diagnosis of MM. Gong et al. (Gong et al., 2021) discovered that circ_0087776 expression was significantly lower in serum of 136 MM patients compared with 74 healthy controls, with the consistent result in MM cells. Importantly, it significantly increased the sensitivity with ALB, β₂-MG joint diagnosis, emphasizing that it could be used as a new biomarker for combined diagnosis of MM (Gong et al., 2021). Altogether, these results verify certain circRNAs as diagnostic and prognostic biomarkers in MM, and further cohort studies are needed to explore more effective biomarkers in the future.

### Molecular mechanisms of circRNAs in MM

**FIGURE 3:**
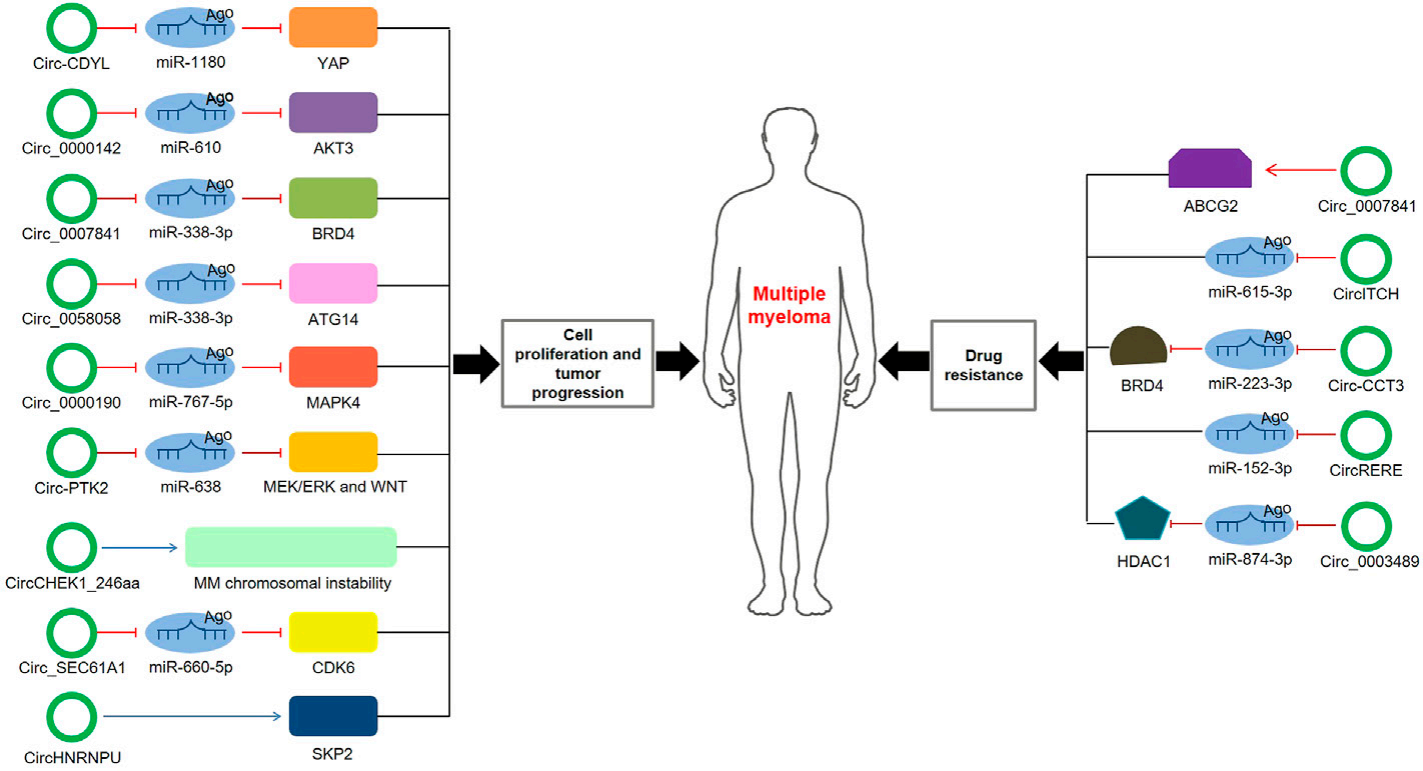
The role and regulatory mechanisms of circRNAs in MM. The schematic diagram depicts the known roles of circRNAs in MM progression and the ways that circRNAs are involved in miRNA-associated gene or gene regulatory pathway.

Although increasing circRNAs are recognized to be promising biomarkers for MM, the related molecular mechanisms remain ambiguous. Here, we highlight several mechanisms of circRNAs in MM (Table 2; Figure 3).

**TABLE 2:** Functional mechanisms of circRNAs in MM.

Classification	CircRNAs	Molecular targets	Functional mechanisms	References
Cell proliferation and tumor progression	Circ-CDYL	miR-1180	Absorbed miR-1180 to upregulate YAP expression	Chen et al. (2020b)
	Circ_0000142	miR-610	Targeted miR-610 and positively regulated AKT3 expression	Liu et al. (2021a)
	Circ_0007841	miR-338-3p	Sponged miR-338-3p and upregulated the expression of BRD4	Wang et al. (2020)
	Circ_0058058	miR-338-3p	Sponged miR-338-3p and positively regulated ATG14 expression	Xue et al. (2021)
	Circ_0000190	miR-767-5p	Repressed miR-767-5p and upregulated MAPK4	Feng et al. (2019)
	Circ-PTK2	miR-638	Sponged miR-638 and activated MEK/ERK and WNT signaling pathways	Zhou et al. (2021)
	CircCHEK1_246aa	-	Increased MM chromosomal instability	Gu et al. (2021)
	Circ_SEC61A1	miR-660-5p	Sponged miR-660-5p to increase CDK6 expression	Luo et al. (2021)
	CircHNRNPU	-	Regulated alternative splicing of SKP2 and increased its expression	Tang et al. (2022)
Drug resistance	Circ_0007841	ABCG2	Upregulated ABCG2 and enhanced MM chemoresistance	Song et al. (2020)
	CircITCH	miR-615-3p	Sponged miR-660-5p to enhance the sensitivity of MM cells to BTZ	Liu et al. (2020a)
	Circ-CCT3	miR-223-3p	Sponged miR-223-3p and increased BRD4 expression	Liu et al. (2022)
	CircRERE	miR-152-3p	Sponged miR-152-3p and facilitated the resistance of MM to BTZ	Fang et al. (2021)
	Circ_0003489	miR-874-3p	Sponged miR-874-3p and positively regulated HDAC1	Tian et al. (2021)
	circPVT1	-	Facilitated the proliferation of MM cells and enhanced glucocorticoid resistance	Wan et al. (2017)

*CircRNAs*, Circular RNAs; *MM*, multiple myeloma *YAP*, yes-associated protein; *MAPK4*, mitogen-activated protein kinase 4; *ABCG2*, ATP, binding cassette transporters G2; *BTZ*, bortezomib.

#### Cell proliferation and tumor progression

Through MM cohort study and the xenograft tumor model, Chen et al. (Chen et al., 2020b) confirmed that circ-CDYL was elevated in tissue and plasma samples of MM patients; mechanically, circ-CDYL could absorb miR-1180 to upregulate yes-associated protein (YAP) expression, ultimately triggering MM progression. Circ_0000142 was also upregulated in MM patients, and its high expression notably enhanced MM cell proliferation, migration and invasion, while suppressed cell apoptosis (Liu F. et al., 2021). Additionally, circ_0000142 directly targeted miR-610 and positively regulated AKT3 expression, consequently enhancing the proliferation and metastasis of MM cells (Liu F. et al., 2021). Analogously, circ_0007841 was found to be highly expressed in MM patients and MM cell lines, and related mechanistic research demonstrated that circ_0007841 upregulated the expression of BRD4 by sponging miR-338-3p, restraining the apoptosis of MM cells and accelerating the progression of MM (Wang et al., 2020). Xue et al. (Xue et al., 2021) investigated that circ_0058058 was highly expressed in MM bone marrow aspirates and cells, and further *in vivo* and *in vitro* experiments showed that circ_0058058 acted as a sponge for miR-338-3p to target and positively regulate ATG14 expression, promoting MM cell proliferation, metastasis and angiogenesis. In contrast, circ_0000190 was downregulated in both peripheral blood and bone marrow tissue; further in-depth mechanistic experiments explored that circ_0000190 repressed miR-767-5p, subsequently targeting and regulating mitogen-activated protein kinase 4 (MAPK4), which contributed to inhibiting MM progression (Feng et al., 2019). A previous cohort study observed that circular RNA protein tyrosine kinase 2 (circ-PTK2) was associated with unfavorable prognosis in MM patients (Zhou et al., 2020). Mechanically, circ-PTK2 reversely regulated miR-638 expression, which activated MEK/ERK and WNT signaling pathways, thus promoting MM cell proliferation and migration (Zhou et al., 2021). Strikingly, *in vitro* and *in vivo* study demonstrated that CHEK1 promoted MM cellular proliferation and evoked drug-resistance partially by increasing MM chromosomal instability (Gu et al., 2021). Besides, circCHEK1_246aa, a circular CHEK1 RNA, was secreted by MM cells and was proved to increase the invasive potential of MM cells (Gu et al., 2021). A current study manifested that circ_SEC61A1 level was increased in MM tissues and cells, which could aggravate MM progression at least partially by regulating miR-660-5p/CDK6 axis (Luo et al., 2021). Further evidence confirmed that circ_SEC61A1 sponged miR-660-5p to increase CDK6 expression, promoting the proliferation and metastasis and restraining the apoptosis of MM cells (Luo et al., 2021). Recently, circHNRNPU, encoding a novel protein named as circHNRNPU_603aa, was associated with poor outcomes in four independent MM patient cohorts, and overexpressed circHNRNPU_603aa promoted MM cell proliferation by regulating the bone marrow microenvironment and alternative splicing (Tang et al., 2022). These results collectively indicate that aberrant circRNA expression is involved in MM cell proliferation, migration, invasion and apoptosis, ultimately affecting cell differentiation and progression.

#### Drug resistance

Drug resistance to chemotherapeutic drugs has become an inevitable phenomenon for the clinical treatment of MM. Mounting evidence has concerned the role of circRNAs in the chemoresistance of MM. Song et al. (Song et al., 2020) disclosed that silence of circ_0007841 could reduce the half-maximal inhibitory concentration and chemoresistance in doxorubicin-resistant MM cells; inversely, overexpression of circ_0007841 could upregulate the ATP-binding cassette transporters G2 (ABCG2) messenger RNA, thus inducing chemoresistance in doxorubicin-resistant MM cells, suggesting that circ_0007841 enhanced MM chemoresistance through upregulating ABCG2. Bortezomib (BTZ), a proteasome inhibitor, is the first-line drug for MM chemotherapy. However, its therapeutic effect in MM has been largely impaired due to the acquired chemoresistance (Gonzalez-Santamarta et al., 2020). CircITCH, a novelly identified circRNA, was downregulated in BTZ-resistant MM cells and played a vital role in the development of BTZ resistance in MM (Liu J. et al., 2020). Further mechanistic research explored that circITCH acted as a sponge for miR-615-3p, subsequently enhancing the sensitivity of MM cells to BTZ by miR-615-3p/PRKCD axis (Liu J. et al., 2020). Another research investigated that downregulation of circ-CCT3 enhanced the sensitivity of BTZ-resistant MM cells to BTZ (Liu et al., 2022). Mechanistically, Circ-CCT3 sponged miR-223-3p, thus increasing BRD4 expression and ultimately contributing to BTZ resistance of MM (Liu et al., 2022). In parallel, circRERE was upregulated in BTZ-resistant MM samples and cells, and it positively regulated CD47 by sponging miR-152-3p, facilitating the resistance of MM to BTZ (Fang et al., 2021). Recently, Tian et al. (Tian et al., 2021) found that silencing of circ_0003489 sensitized MM cells to BTZ *in vitro* and *in vivo* experiments. In-depth study unveiled that circ_0003489 sponged miR-874-3p and positively regulated its target protein, HDAC1, therefore attenuating the cytotoxic effects of BTZ in MM cells and reversing its inhibiting effect on autophagy (Tian et al., 2021). Glucocorticoid resistance was another primary factor of refractory relapse in MM patients. Wan et al. (Wan et al., 2017) found that silencing circPVT1 could enhance sensitivity to glucocorticoid treatment and inhibit the proliferation rate of MM models.

In collection, these data suggest that circRNAs play a crucial role in the pathogenesis of MM, and elaborated and comprehensive molecular mechanisms are needed to enhance our understanding of MM.

## CircRNAs as potential therapeutic targets for MM

As their regulatory roles and related molecular mechanisms in MM are gradually being recognized, circRNAs may be developed as effective therapeutic targets. Several therapeutic strategies based on the functions of circRNAs have been proposed for the treatment of MM. Regulation of relevant circRNAs through small interfering RNAs (siRNAs), shRNAs and plasmid vector is the most common methods to reduce the expression and overexpression of circRNAs, as well as regulate miRNA molecules. Of note, siRNAs or shRNAs were used to inhibit circRNAs’ expression by targeting their specific backspliced sequence (Yu et al., 2016). Besides, the RNA targeting CRISPR/Cas13 system could achieve knockout of circRNAs and become a useful tool for the functional study of circRNAs (Abudayyeh et al., 2017; Cox et al., 2017; Li et al., 2021; Gao et al., 2022). On the other hand, plasmid and lentiviral vectors were able to increase circRNA levels (Chen et al., 2017; Yao et al., 2017). Also, sequestering translation initiation sites of dysfunctional mRNAs might reduce disease-associated protein biogenesis by artificially controlling endogenous circularization with the use of “mRNA trap” (Chao et al., 1998). In short, such circRNAs will be promising application for MM treatment.

CircRNAs identified as drugs or targets have attracted extensive attention because mounting evidence proved that exosome-mediated delivery of circRNAs could be used for potential therapeutic targets (Kumar et al., 2015). By performing high-throughput sequencing, Sun et al. (Sun et al., 2021) discovered abnormal expression of serum exosomal circRNAs (exo-circRNAs) in patients with MM-related myocardial damage and abnormal expression of these circRNAs could interfere with MM-related myocardial damage by regulating miRNA and the TLR4 axis, providing a notion that exo-circRNAs might be a novel potential therapeutic target for this disease. Consistently, another recent study identified that aberrant expression of the serum exo-circRNAs was also associated with MM-related peripheral neuropathy and might act as therapeutic target for it (Zhang et al., 2021). Hence, given the importance of these results, future studies on circRNAs as therapeutic targets will become one of the most noticeable areas.

## Conclusion and perspectives

With the development of RNA-Seq and other high-throughput technologies (Fan et al., 2015; Verboom et al., 2019), circRNAs are becoming a novel research field in tumor biology and therapy. Spontaneously, more and more MM-related circRNAs along with biological functions have been explored and exhibit great potential to be biomarker for MM diagnosis and prognosis as well as novel therapeutic targets. In this review, we summarize the biogenesis and biological functions of circRNAs, as well as highlight the current state-of-the-art knowledge regarding the functional mechanisms of circRNAs in MM with a focus on the novel therapeutic targets of MM.

Despite the fact that great advance has been made in the research of circRNAs in MM, there are several aspects of circRNAs need to be addressed before they can be incorporated into clinical practice. First, recent and current studies are simple and limited on exploring circRNAs as MM biomarkers and therapeutics targets or tools. Although the available databases, tools and computational methods dedicated to circRNAs has been investigated (Xiao et al., 2022), more complete databases, particularly those that include information on tissue-specific circRNAs are needed in the near future. Second, a majority of mechanisms of circRNAs are involved in miRNA sponge in MM, while circRNAs mechanisms of circularization and degradation remain poorly understood. Thus, novel mechanisms other than miRNA sponge require to be disclosed. Last, it’s worth noting that exosomal circRNAs can be released by many cells through extracellular vesicles (Kooijmans et al., 2012; Raposo and Stoorvogel, 2013), which can transfer signaling molecules to recipient cells and participate in cancer progression. For instance, exosomal circ-ATP10A acted as a miRNA sponge and regulated the protein levels of downstream vascular endothelial growth factor-B in MM patients (Yu et al., 2022). Additionally, recent research revealed that numbers of circRNAs are enriched in exosome and even acted as effective carriers for siRNAs delivery of gene therapy, which could be a promising biomarker for tumor diagnosis or therapeutic targets (Li Y. et al., 2015; Kumar et al., 2015). Hence, exosomal circRNAs should be studied in detail in future studies and are expected to become remarkable biomarkers and therapeutic targets for MM.

Ultimately, taken in-depth research into the mechanisms of circRNAs underlying MM development and progression will facilitate the optimization of the diagnosis and therapy of MM.

## Abbreviations

ABCG2, ATP binding cas sette transporters G2; BM, bone marrow; BTZ, Bortezomib; circ-PTK2, circular RNA protein tyrosine kinase 2; CircRNAs, Circular RNAs; ciRNAs, circular RNAs; EIciRNAs, exon intron circular RNAs; exo-circRNAs, exosomal circRNAs; f-circRNAs, fused circRNAs; IRES, inserted an internal ribosome entry site; MAPK4, mitogen-activated protein kinase 4; MM, multiple myeloma; mt-circRNAs, mitochondrial genome-derived circRNAs; miRNA, microRNA; ecircRNAs, exonic circRNAs; PES1, pescadillo homologue 1; PFS, progression-free survival; OS, overall survival; RBP, RNA binding proteins; siRNAs, small interfering RNAs; tricRNAs, rt-circRNAs, read-through circRNAs; tRNA intronic circRNAs; YAP, yes-associated protein.
